# Exploring Graphene and MoS_2_ Chips Based Surface Plasmon Resonance Biosensors for Diagnostic Applications

**DOI:** 10.3389/fchem.2020.00728

**Published:** 2020-08-26

**Authors:** Devi Taufiq Nurrohman, Ying-Hao Wang, Nan-Fu Chiu

**Affiliations:** ^1^Laboratory of Nano-photonics and Biosensors, Institute of Electro-Optical Engineering, National Taiwan Normal University, Taipei, Taiwan; ^2^Department of Electronics Engineering, State Polytechnic of Cilacap, Cilacap, Indonesia; ^3^Department of Life Science, National Taiwan Normal University, Taipei, Taiwan

**Keywords:** surface plasmon resonance, biosensor, 2D materials, graphene, diagnostic, MoS_2_

## Abstract

Until now, two-dimensional (2D) nanomaterials have been widely studied and applied in the biosensor field. Some of the advantages offered by these 2D materials include large specific surface area, high conductivity, and easy surface modification. This review discusses the use of 2D material in surface plasmon resonance (SPR) biosensor for diagnostic applications. Two-dimensional material reviewed includes graphene and molybdenum disulfide (MoS_2_). The discussion begins with a brief introduction to the general principles of the SPR biosensor. The discussion continues by explaining the properties and characteristics of each material and its effect on the performance of the SPR biosensor, in particular its sensitivity. This review concludes with some recent applications of graphene- and MoS_2_-based SPR biosensor in diagnostic applications.

## Introduction

The main challenge for all electrical, mechanical, and optical sensors is to detect chemical and biological analytes with low molecular weight (<400 Da) in very dilute conditions (Guo and Tan, 2009). Since surface plasmon resonance (SPR) biosensor was first introduced in the early 1990s, it has proven to be one of the most powerful technologies for determining specificity, affinity, and kinetic parameters during binding of macromolecules in many types of bonds, including protein–protein (Kim et al., 2006), protein–DNA (Majka and Speck, 2006), enzyme–substrate or inhibitor (Fong et al., 2002), receptor drug (Rich et al., 2002), lipid membrane–protein (Erb et al., 2000), protein–polysaccharide (Beccati et al., 2005), and cell– or virus–protein (Zhang et al., 2014), among others. One of the advantages offered by this device is its unique ability to monitor molecular binding activity in real time (Zeng et al., 2014).

The SPR biosensor is a type of biosensor that is very sensitive to changes in the refractive index on the SPR sensing surface. The working principle of the SPR biosensor is based on the collective coherent oscillation of free electrons in the metal conduction band first excited by the interactive electromagnetic field at the metal/dielectric interface, and the created charge density oscillation is called surface plasmon polaritons (SPPs) (Raether, 1988). The SPPs will then form an electric field that exponentially decays into the surrounding media with a depth of penetration in the range of hundreds of nanometers. As a result, this evanescing electric field is very sensitive to changes in the surrounding refractive index. Thus, when there is a change in the refractive index of the medium, the characteristics (e.g., angle, wavelength, phase, etc.) of the light beam for SPR excitation will also change (Zeng et al., 2014).

There are several metals that can be used to excite SPPs including gold (Au), silver (Ag), copper (Cu), aluminum (Al), sodium (Na), and indium (In) (Raether, 1988; Maurya and Prajapati, 2016). Na is reactive in nature, In is very expensive, whereas Ag, Cu, and Al are susceptible to oxidation; in contrast, Au is resistant to oxidation and corrosion in different environments. Therefore, Au is the best choice as an active metal in conventional SPR sensor. However, bare Au surfaces are not suitable for the biosensor because of its poor absorbance properties of biomolecules (Wu et al., 2010). Therefore, traditional biosensors are not capable to detect low molecular weight of biomolecules because of poor attachment of these biomolecules to the bare metal surface (Maurya and Prajapati, 2016). Until now, many methods have been developed to increase the sensitivity of SPR sensor such as using adhesion layer (Agarwal et al., 2016a,b), metal nanoparticles and nanohole (Prasad et al., 2019), metal nanoslits (Yeung et al., 2018), and gold nanoparticles (Amendola et al., 2017). But until now, precise control over the geometry and optical properties of these nanostructures is still very challenging (Kasani et al., 2019).

Recently, there have been many publications on SPR biosensors that use thin films with high refractive index [Si and two-dimensional (2D) materials such as graphene and molybdenum disulfide (MoS_2_)] to increase sensor sensitivity (Tabasi and Falamaki, 2018). Two-dimensional materials such as graphene and MoS_2_ have unique properties and offer promising opportunities. Graphene has a high surface-to-volume ratio, which will produce strong interactions with biomolecules, excellent transparency, electron conductivity, and superior mobility (>2 × 10^5^ cm^2^ V^−1^ s^−1^ at electron density 2 × 10^11^ cm^−2^), large specific surface area (>2,500 m^2^ g^−1^), large Young's modulus (>0.5–1 TPa), and high thermal conductivity (>3,000 W mK^−1^) (Wang et al., 2019). Besides graphene, MoS_2_ is another 2D material that has recently been used for SPR applications. This material has higher optical absorption than graphene with exceptional optical and electrical properties (Ouyang et al., 2016). More importantly, the cytotoxicity and genotoxicity of MoS_2_ are considered relatively low to most biospecies, which is the pre-requisite for the applications in biosensing (Kaur et al., 2018; Hu et al., 2019).

Many researchers report that individual graphene and MoS_2_ can increase the sensitivity of SPR through a simulation approach. Verma et al. (2015) reported that by adding graphene to the gold surface, the sensitivity of the sensor had increased from 30.85 to 33.98°/(RIU: refractive index unit). Similar results were also reported by Maurya and Prajapati (2016). They compared the sensitivity of SPR on four different structures, namely, bare Ag, Ag/graphene, Ag/MoS_2_, and Ag/MoS_2_/graphene. At the change of the refractive index of 0.068, the SPR angle shifts in the four structures were 4.38, 4.41, 4.56, and 4.61°. Based on these results, MoS_2_ individuals showed better sensitivity than graphene. But the best performance is in the structure composed of MoS_2_ and graphene (Maurya and Prajapati, 2016). Similar results were also shown by other metals such as Cu and Au (Maurya et al., 2015; Zeng et al., 2015; Maurya and Prajapati, 2016). In addition, some researchers also claim that 2D materials such as graphene can protect reactive metals (Cu, Ag, etc.) for a long time (about 1 year) in the air and water environment (Kravets et al., 2014) because of its nature, which is impenetrable to most atoms and ions (Geim and Novoselov, 2007). This is very important for the purpose of maintaining the quality factor during the functionalization process and biomolecular detection (Wu et al., 2019).

In this review, the authors summarize the current development of 2D nanomaterials, namely, graphene and MoS_2_ in SPR biosensor. The discussion begins by discussing the general principles of the SPR biosensor. The discussion continues by explaining the properties and characteristics of each material and its effect on the performance of the SPR biosensor, in particular its sensitivity. This review concludes with some recent applications of graphene- and MoS_2_-based SPR biosensor in diagnostic applications.

## General Principle of SPR Biosensor

**Figure 1:**
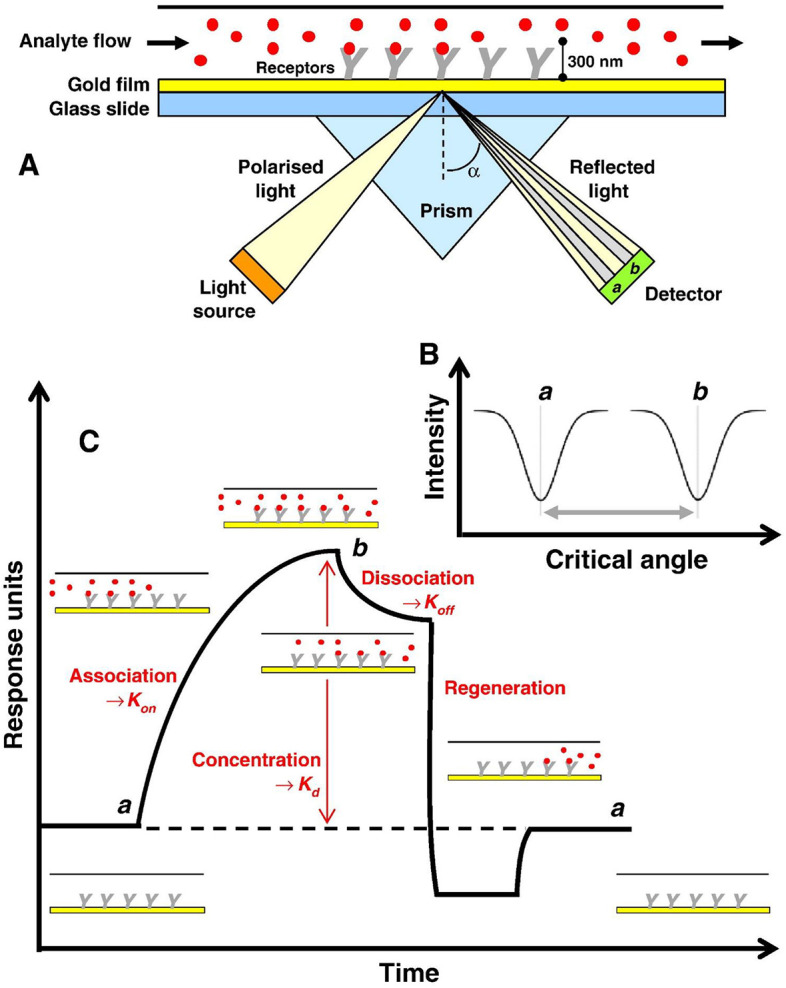
**(A)** Instrument setup for an SPR experiment. **(B)** Change in the SPR angle of incident light from angle a to angle b on the binding of an analyte molecule to a bioreceptor molecule. **(C)** Response of the SPR experiment in the form of a sensogram. Figures **(A–C)** were reproduced from Patching (2014) with permission from Elsevier. Copyright 2014, Elsevier.

There are several types of SPR biosensor platforms. Some of them are attenuation-total reflection, optical prism couplings, optical fiber couplings, grating couplings, and others. Of the many platforms, the prism coupling based on Kretschmann configuration has become standard technique to excite SPPs (Prabowo et al., 2018). In this configuration, the metal is usually deposited on the surface of the prism. After that, the prism is illuminated by light which is *p*-polarized with a certain angle of incidence (Figure 1A) (Damborský et al., 2016). By changing the angle of incidence, a sharp decrease is found in the intensity of reflected light for certain range of incident angles. The angle at which the minimum reflected light is called the SPR angle, which in theory can be determined by the equation:

$$\begin{array}{l} \theta_{SPR}=sin^{-1}\left(\frac{1}{n_{1}}\sqrt{\frac{n_{2}^{2}n_{m}^{2}}{n_{2}^{2}+n_{m}^{2}}}\right) \end{array}$$

where θ_*SPR*_ shows the SPR angle; *n*_1_ and *n*_*m*_, respectively, indicate the refractive indices of prism and metal. Adsorption and desorption occurring on metal surfaces change the refractive index of the near media metal–dielectric interface and change the SPR angle (Figure 1B). Therefore, monitoring of changes in the SPR angle can be used to analyze adsorption–desorption activities or associations that occur on metal surfaces (Tang and Zeng, 2010).

Monitoring of adsorption and desorption activities on the metal surface of the SPR is expressed in a curve called a sensogram as shown in Figure 1C. Sensogram can be obtained based on changes in SPR angle or changes in reflectivity at any time due to biomolecular interactions (Schasfoort, 2008). The monitoring process using this sensogram begins with the sensor surface conditioning using an appropriate buffer solution to create a baseline and activate a ligand that functions as a bioreceptor to capture the target analytes. The next step is to inject the analyte onto the sensing surface. The target molecule will be selectively captured by the ligand. The more molecules captured, the higher the SPR angle/reflectivity changes. Next, a buffer is injected into the sensor and the unattached component specifically flows during the dissociation phase. In this step, also the dissociation phase of the analyte begins. Finally, a regeneration solution is injected to break the specific bond between the analyte and the ligand. If ligands are properly immobilized on the sensor surface, they remain on the sensor, whereas the target analytes are removed quantitatively (Ritzefeld and Sewald, 2012; Patching, 2014). Based on this sensogram curve, several parameters can be obtained including the association rate constant (*K*_on_), the dissociation rate constant (*K*_off_), and the equilibrium dissociation constant (*K*_d_) (Moscetti et al., 2017).

## Graphene-Based SPR Biosensor

### Graphene Properties and Their Potential in the SPR Biosensor

Graphene is the name of a single layer of carbon atoms arranged in a 2D crystalline hexagonal lattice due to the sp^2^ hybridization of carbon. Thus, graphene has strong in-plane σ bonds, responsible for its high mechanical strength and flexibility, and it also has weak out-of-plane π bonds responsible for its thermal carrying, electrical charge, and transparency (Amieva et al., 2016). When compared with conventional noble metals such as Au, Ag, Cu, Cr, and Al, graphene has low energy losses (e.g., Ohmic loss and radiative loss) and good tunability. The confinement of the surface plasmons (SPs) in the graphene is much stronger than in conventional noble metals (Luo et al., 2013). All of these advantages make graphene a promising material for future sensor applications. Recently, graphene has emerged as an alternate plasmonic material but only in the terahertz to mid-infrared range (Gupta et al., 2019).

In SPR biosensor, a plasmonic metal that is functionalized with graphene has four advantages, namely, (i) graphene has a very high surface-to-volume ratio, which is expected to be beneficial for efficient adsorption of biomolecules compared with bare metal; (ii) graphene increases the adsorption of organic and biological molecules because their carbon-based ring structure enables π stacking interaction with the hexagonal cells of graphene; (iii) controlling the number of graphene layers transferred on to the plasmonic metal interface enables control of the SPR response and the sensitivity of SPR measurements (Szunerits et al., 2013); (iv) the presence of graphene on top of plasmonic metal can be used to protect metals from oxidation so that the stability and quality factor of plasmonic metal can be maintained (Szunerits et al., 2013; Kravets et al., 2014; Wu et al., 2019).

Graphene has high electron transport mobility and a high surface-to-volume ratio. Electrons move at a speed of 1 million meters per second (highest mobility ~ 200,000 cm^2^/Vs). This property makes it possible to be a future sensor with ultrafast speed. Because this is a 2D material, each graphene atom can be considered a surface atom, and as a result, each atomic site can be involved in biomolecular interactions. This graphene feature can ultimately be responsible for the response of ultrasensitive sensors with the lowest detection capability to even one single molecule (Basu and Bhattacharyya, 2012; Guy and Walker, 2016). The use of pristine graphene has proved challenging because of difficult bottom-up synthesis (Smith et al., 2019), poor solubility (Yan, 2018), and agglomeration in solution due to van der Waals interactions (Skoda et al., 2014). As an alternative, compounds similar in structure to graphene can be synthesized from graphite or other carbon sources by a top-down method in an effort to achieve many of the advantages of pristine graphene while also imbuing the surface with functionalized oxygen groups. The oxidation of graphite in protonated solvents leads to graphite oxide, which consists of multiple stacked layers of graphene oxide (GO) (Smith et al., 2019).

Graphene oxide has a hexagonal carbon structure similar to graphene but also contains hydroxyl (–OH), alkoxy (C–O–C), carbonyl (C–O), carboxylic acid (–COOH), and other oxygen-based functional groups. This oxygenated group is responsible for many advantages over graphene, including higher solubility and the possibility of easier surface functionalization with various types of bioreceptors. Several studies have reported that GO is compatible with single-strain DNA (ssDNA), peptides, and amino acids (Sharma et al., 2016). Through thermal, chemical, and electrochemical treatments, the oxygen functional groups in GO can be reduced to produce reduced GO (rGO). In rGO, the number of oxygen function groups is less than GO. Reduced GO can be considered as an intermediate structure between a pristine graphene and a highly oxidized GO, thus retaining some and losing some of the other properties of the two materials (Reina et al., 2017; Banerjee, 2018). Their interlayer distance was reduced from 7.9 Å on GO to 3.4 Å on rGO (Kitayama et al., 2019). By controlling the ratio of carbon to oxygen and the chemical composition in rGO, this material can be alternative for biological and biosensor applications. This can be done by selecting the reduction method in accordance with the expected properties (Pei and Cheng, 2012; Banerjee, 2018).

### Effect of Graphene on SPR Sensitivity

Sensitivity can be defined based on the value of the limit of detection and the linearity of the biosensor. Limit of detection is very important because it shows the smallest concentration that can be detected by biosensors. In many cases today, biosensors are required to be able to detect biomolecules in the concentration range of ng/mL or fg/mL (Zagorodko et al., 2014; Maurya and Prajapati, 2020). For example, in the case of prostate cancer, the prostate-specific antigen has a concentration of 4 ng/mL in the blood. The development of biosensors is currently leading to efforts to achieve sensors that are as sensitive as possible (Metkar and Girigoswami, 2019).

In the SPR biosensor, the electromagnetic field is an evanescent wave that decays exponentially into both the metal and sensing layer regions. An evanescent electromagnetic field enhancement leads to the increase in the SPR sensor sensitivity to perturbations in the sensing layer refractive index. In addition, the increase in the sensing layer refractive index causes the increase in the SPR sensor sensitivity (Tabasi and Falamaki, 2018). Shalabney and Abdulhalim (2010) investigated the effect of material with a high refractive index (silicon) with a thickness of 10.5 nm, which was deposited on a metal surface. The results obtained indicate that the presence of silicon increases the intensity of evanescent electromagnetic fields and SPR sensitivity. The sensitivity of SPR produced on structures with and without silicon are 200 and 67.5°/RIU, respectively (Shalabney and Abdulhalim, 2010). Maharana et al. (2015) compared the increase in evanescent electromagnetic fields in silicon (10 nm) and monolayer graphene (0.34 nm) deposited in silver (43 nm). The fields produced on the surfaces of silicon and graphene monolayer are 375 and 825 (A/m)^2^, respectively. In addition, at a wavelength of 653 nm, the SPR sensitivity on graphene is 340% higher than silicon. Based on the above data, it can be concluded that graphene in the SPR biosensor has a significant role in increasing the evanescence electromagnetic fields and SPR sensitivity (Maharana et al., 2015).

**Figure 2:**
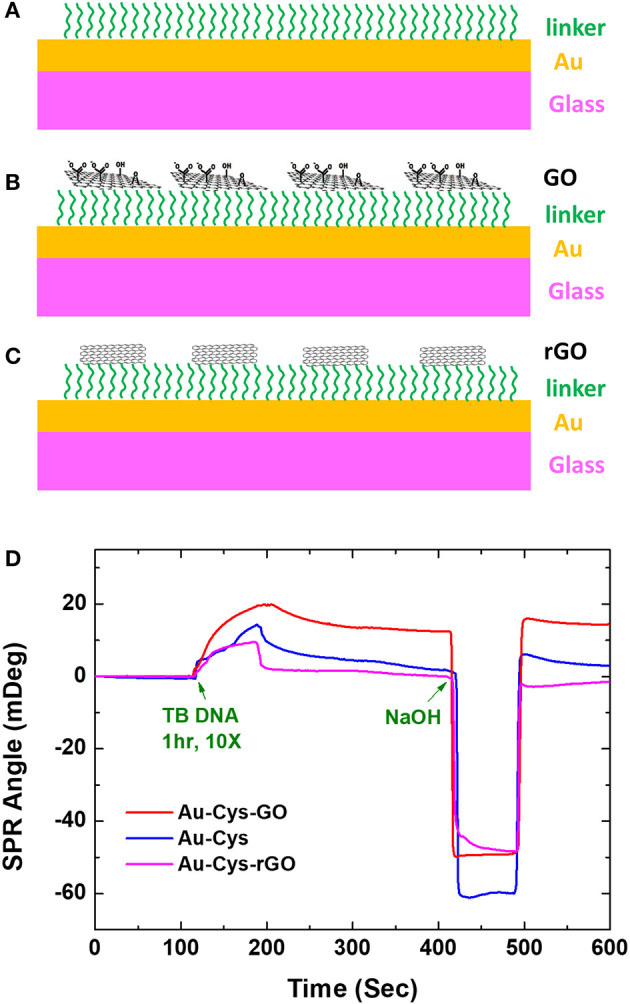
Three fabricated chips. **(A)** Conventional chip. **(B)** GO-SPR chip. **(C)** rGO-SPR chip. **(D)** SPR sensogram on conventional chip, GO-SPR chip, and rGO-SPR chip. Figures **(A–D)** were reproduced from Chiu et al. (2012) with permission from the SPIE. Copyright 2012, SPIE.

Research on graphene-based SPR biosensor in the group led by Professor Chiu has been started since 2012. Preliminary research in this group is to investigate SPR biosensor on single-layer GO and rGO to detect tuberculosis bacterial DNA (TB DNA). The study was carried out by investigating three different chips, namely, GO-based SPR chip (GO-SPR), rGO-based SPR chip (rGO-SPR), and conventional SPR chip. The cystamine dihydrochloride (Cys) ring was deposited on the gold surface to detect TB DNA on conventional chip and to immobilize GO and rGO sheet on SPR chips (Figures 2A–C). Figure 2D is an SPR sensogram that shows the SPR response after TB DNA is injected into each chip. Based on the spectrum produced, the Au-Cys-GO chip shows the highest SPR angle shift. Surface plasmon resonance angle shifts on GO-SPR, rGO-SPR, and conventional SPR chips are 15,646, 2,312, and 5,418 mDeg, respectively. After NaOH is injected, the baseline on the GO-SPR chip is not reduced. This shows a very strong covalent bond between the surface of Au-Cys-GO and TB DNA (Chiu et al., 2012).

**Figure 3:**
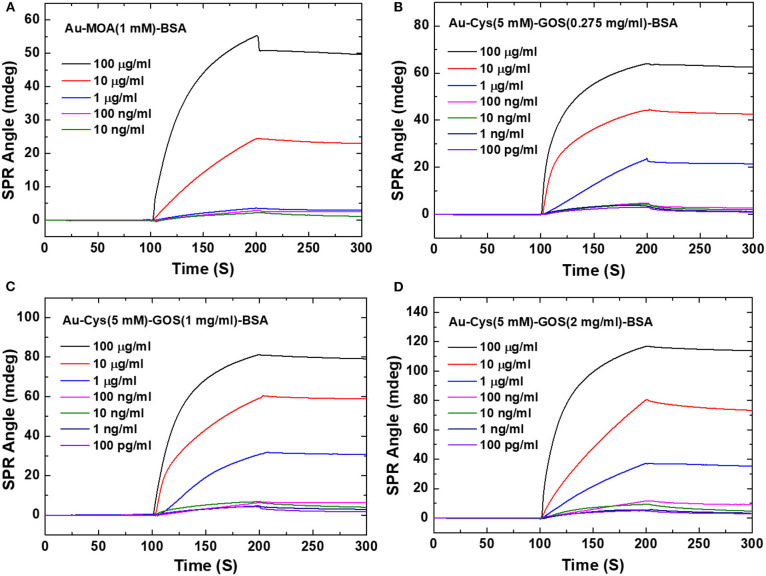
Surface plasmon resonance sensograms obtained in response to BSA solutions of different concentrations flowing over the surfaces of the sensors. **(A)** Interaction with the conventional Au film-based (Au–MOA) sensor (1 mM). **(B)** Interaction with the 0.275 mg/mL GOS. **(C)** Interaction with the 1 mg/mL GOS sensor. **(D)** Interaction with the 2 mg/mL GOS sensor. Figures **(A–D)** were reproduced from Chiu and Huang (2014) with permission from Elsevier. Copyright 2014, Elsevier.

Based on the results of the above study, Chiu and Huang (2014) further performed GO variations to detect immobilization of bovine serum albumin (BSA). Graphene oxide with concentrations of 0.275, 1, and 2 mg/mL were immobilized on the gold surface using cystamine linker. Furthermore, the carboxyl group on GO is activated using 1-ethyl-3-(3-dimethylaminopropyl) carbodiimide (EDC) and *N*-hydroxysuccinimide (NHS) with a concentration ratio of 4:1. The results obtained indicate that all fabricated chips can detect BSA directly. At BSA concentrations of 100 pg/mL−100 μg/mL, the SPR sensogram shows that the higher the BSA concentration, the higher the SPR angle (Figure 3). The highest sensitivity found on SPR chips with GO concentrations is 2 mg/mL. At this concentration, the SPR chip can detect BSA up to a concentration of 100 pg/mL, which is 4.3 times greater when compared to conventional chips [Au–mercaptooctanoic acid (MOA)].

**Figure 4:**
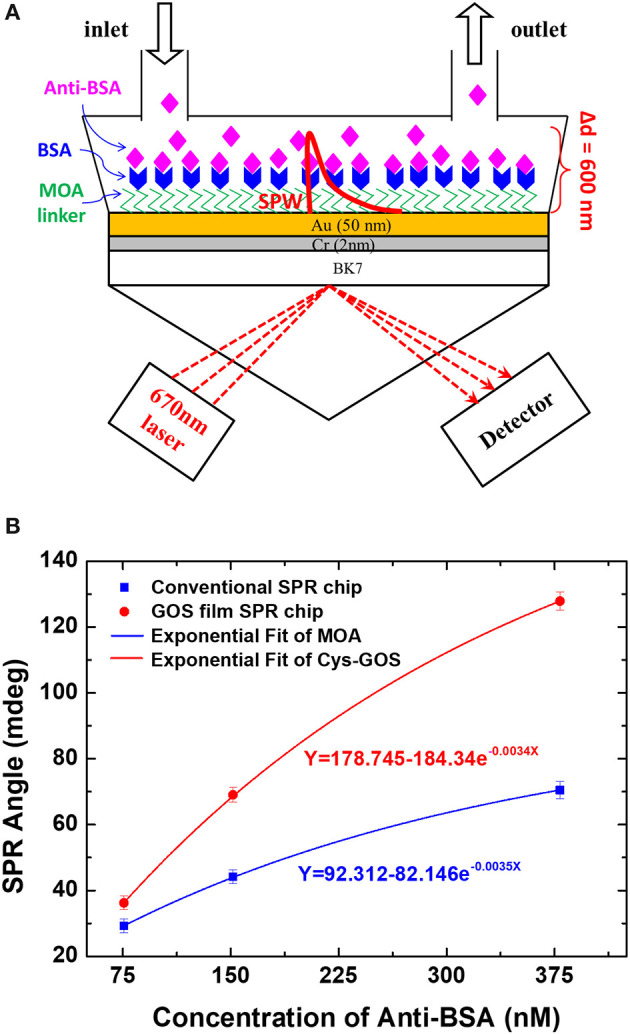
**(A)** SPR experimental scheme to study the interaction of BSA and anti-BSA. **(B)** Equilibrium analysis of binding of anti-BSA and BSA. Figures **(A,B)** were reproduced from Chiu et al. (2014) with permission from the Nanoscale Research Letters. Copyright 2014, Springer Nature.

In the same year, Chiu et al. (2014) applied a GO-based SPR biosensor that had been developed previously to study the interaction of antibody and antigen. In this study, they studied the interaction between BSA and anti-BSA. The study was conducted by comparing GO-SPR chip and conventional chip that had been developed previously. Based on Figure 4, the SPR angle shift at the anti-BSA concentration of 75.75 nM on the GO-SPR chip is 1.4 times higher than conventional chip. At the highest concentration (378.78 nM), the change in SPR angle on GO-SPR chip is two times higher than conventional chip. This shows that GO-SPR chip is more sensitive than conventional chip so that this GO-SPR chip has the potential to be used in clinical diagnoses with lower concentrations.

**Figure 5:**
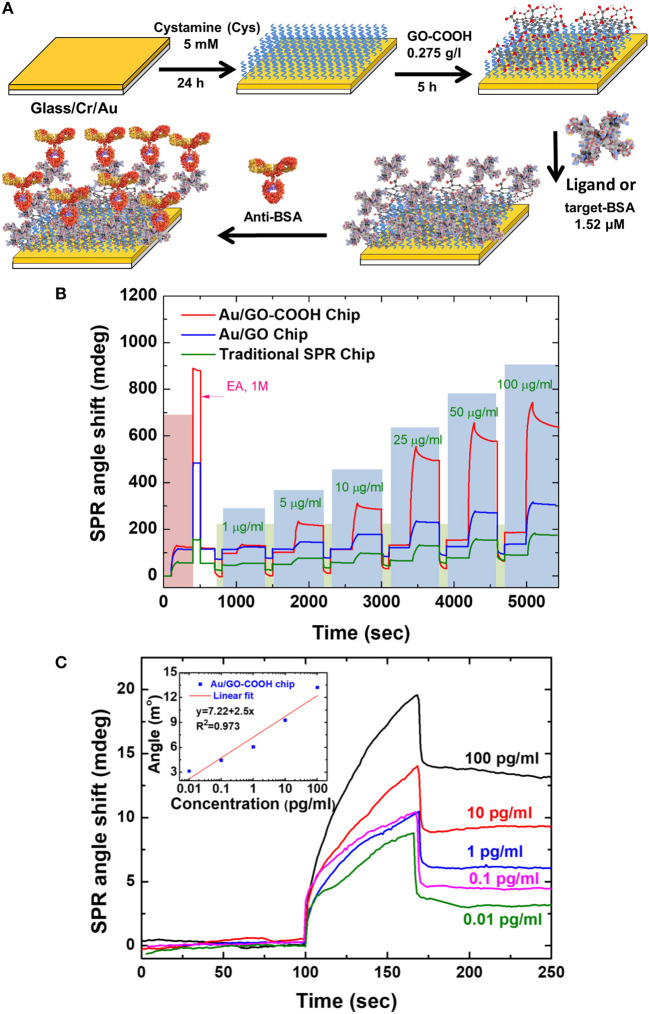
**(A)** Fabrication of SPR chip with biomolecular immobilization on modified surface of carboxyl-functionalized GO film. **(B)** Analysis of antigen-antibody interaction on various sensing chips with various analyte concentrations. **(C)** Sensogram and calibration curve on Au/GO-COOH chip at concentrations of 0.01–100 pg/mL. Figures **(A–C)** were reproduced from Chiu et al. (2017a) with permission from Elsevier. Copyright 2017, Springer Nature.

In 2016, Chiu et al. (2017a) modified the GO-SPR chip that was developed previously by adding the carboxyl group (–COOH) to the SPR chip to study the anti-BSA and BSA interactions. The immobilization procedure on the SPR chip and the results obtained are shown in Figures 5A–C. Based on Figure 5B, the SPR angle shift at the anti-BSA concentration of 1–100 μg/mL shows that the higher the anti-BSA concentration, the higher the SPR angle shift. Of the three chips fabricated, the Au/GO-COOH chip shows a higher response so that this chip has the best sensitivity. The initial conclusion of this experiment is that the presence of carboxyl groups on GO surfaces greatly influences the performance of the SPR biosensor. Authors tried to reduce the anti-BSA concentration to a concentration of 0.01 pg/mL. Linear curves are obtained when the anti-BSA concentration is 0.01–100 pg/mL. The results of this study indicate that GO-SPR chips modified with the carboxyl group have the best performance when compared with previous studies (Chiu et al., 2017a).

### Current Application of Graphene-Based SPR Biosensor

**Figure 6:**
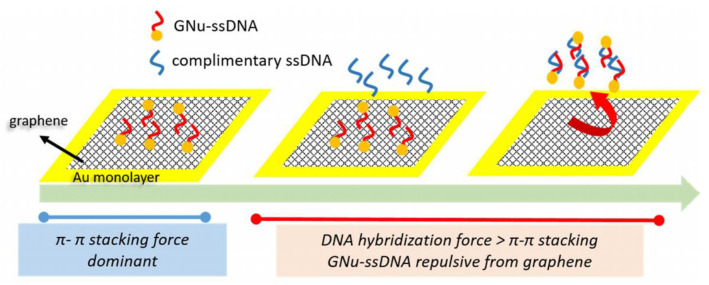
The cssDNA detection mechanism developed by Prabowo et al. This figure was reproduced from Prabowo et al. (2016) with permission from Elsevier. Copyright 2016, Elsevier.

Prabowo et al. (2016) developed a graphene layer to investigate DNA hybridization of *Mycobacterium tuberculosis* using the SPR biosensor. The detection mechanism is shown in Figure 6. The graphene layer is deposited on the SPR chip using the simple drop casting method. Furthermore, an ssDNA binds covalently to gold nano urchin (GNu) and forms a sensing probe called ssDNA–GNu. The binding mechanism of the graphene and ssDNA layers is caused by the existence of the π*-*π stacking force. When hybridization occurs between complementary ssDNA (cssDNA) and ssDNA, the hybridization force is more dominant than the π*-*π stacking force. The presence of cssDNA will disrupt the ssDNA–GNu from the graphene layer. The detection limit achieved from this experiment was 28 fM (Prabowo et al., 2016).

**Figure 7:**
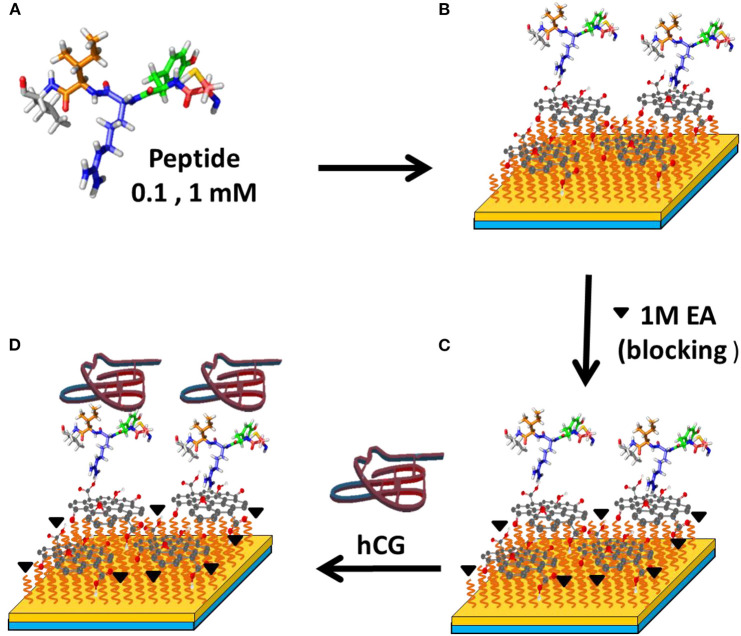
Surface modification to detect hCG protein with SPR biosensor. Figures **(A–D)** were reproduced from Chiu et al. (2017b) with permission from Elsevier. Copyright 2017, Elsevier.

In 2017, Chiu et al. (2017b) combined GO sheets with specific peptide aptamer to detect human chorionic gonadotropin (hCG) proteins. The surface functionalization procedure for GO with peptide is shown in Figure 7. In this study, authors used (N-) PPLRINRHILTR (-C) (N-Pro-ProLeu-Arg-Ile-Asn-Arg-His-Ile-Leu-Thr-Arg-C) to assay hCG protein. To block potential sites of interaction, the remaining carboxyl groups that are activated on the surface of the GO sheet are blocked by injecting ethanolamine solution. From this experiment, the limit of detection obtained was 0.065 nM with sensitivity 16 times higher than conventional SPR chips.

**Figure 8:**
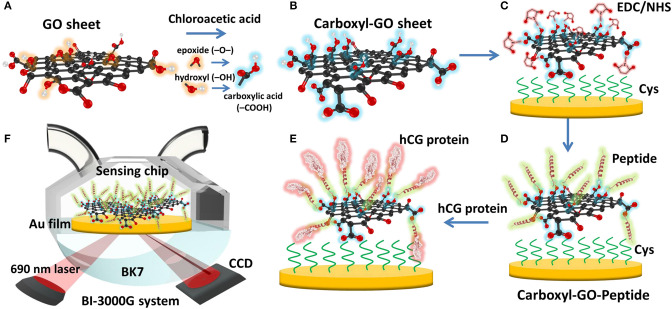
Schematic illustration of the conversion of **(A)** GO into **(B)** carboxyl-GO sheets via a facile one-step chloroacetic acid modification route. **(C)** GO surface activation with EDC/NHS. **(D)** The attachment of peptides via amine coupling and the deactivation of the unreacted surface sites. **(E)** Immobilization of the peptide on the carboxyl-GO–based SPR chip using non-immunological to detect hCG protein. **(F)** Schematic instrumental setup of the Kretschmann configuration. Figures **(A–F)** were reproduced from Chiu et al. (2019a) with permission from Dove Medical Press.

In 2019, Chiu et al. (2019a) detected the same protein (hCG) using a modified GO-based SPR chip by adding a carboxyl group to the sensing surface (Figure 8). Based on the results of previous studies, the carboxyl group on the sensing surface produces high affinity and stronger binding of biomolecules. To test biosensor selectivity, hCG protein was mixed with 20 nM BSA and 20 nM Human serum albumin (HSA). Based on the calibration curve, there is no significant interaction between peptides with BSA and HSA. This shows high selectivity and a strong bond between peptides and hCG. The limit of detection for hCG in clinical serum samples is 1.15 pg/mL.

**Figure 9:**
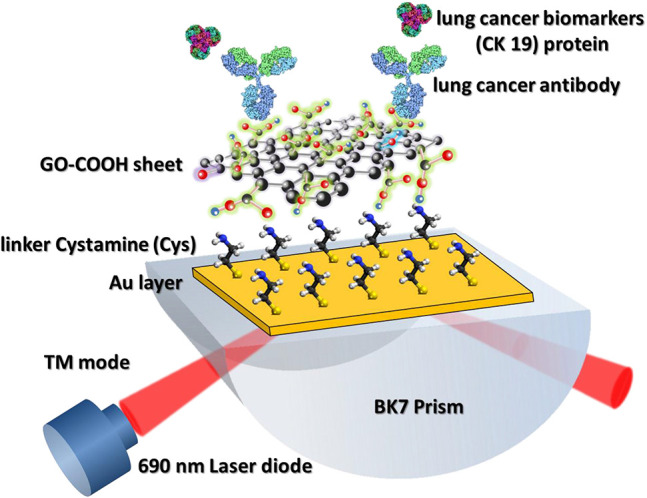
Graphene oxide–COOH sheet-based SPR immunosensor to detect CK19 protein. The figure was reproduced from Chiu et al. (2018) with permission from Elsevier. Copyright 2018, Elsevier.

Chiu et al. (2018) also used carboxyl-GO–based SPR immunosensor to detect non–small cell lung carcinoma through cytolerayin 19 (CK19) protein biomarkers in spiked human plasma. In this immunosensor, small amounts of CK19 specific antibodies are immobilized on the SPR chip to specifically detect CK19 protein (Figure 9). Next, CK19 protein with different concentrations is injected into a functioning SPR chip. Based on the SPR angle response at each CK19 protein concentration, it was concluded that the SPR immunosensor had good linearity at a CK19 concentration of 0.001–100 pg/mL (Chiu et al., 2018). These results reaffirm that the presence of carboxy groups on the GO surface has been shown to increase biosensor sensitivity.

**Figure 10:**
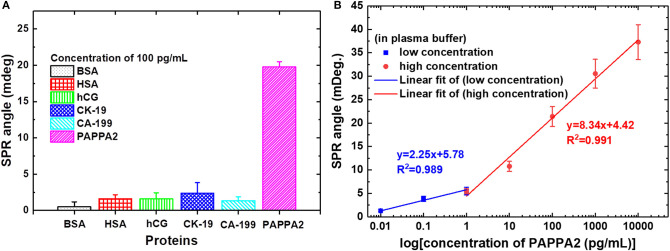
**(A)** The SPR angle shift plot of six different proteins to determine biosensor selectivity. **(B)** Calibration curve of the average SPR response to various PAPP-A2 protein concentrations. Figures **(A,B)** were reproduced from Chiu et al. (2019b) with permission from the Dove Medical Press.

In 2019, Chiu et al. (2019b) developed a carboxyl-GO–based SPR immunosensor to detect pregnancy protein-associated plasma protein A2 (PAPP-A2) in human blood plasma. The carboxyl-GO surface was functionalized by utilizing covalent bonds between carboxylic acid and anti–PAPP-A2 protein. In addition, BSA was covalently immobilized to block carboxyl-GO sheets in areas that are not coated with anti–PAPP-A2 protein. Figure 10A shows how the sensor selectivity is generated. Of the six types of proteins that are injected on the sensing surface, the highest SPR angle shift lies in the PAPP-A2 protein. The SPR angle shift in other types of proteins is much smaller. Therefore, it can be concluded that the SPR immunosensor developed has good selectivity. After that, the accuracy and precision of the GO-carboxyl–based SPR chip are tested based on the SPR angle response at different concentrations of PAPP-A2 protein (Figure 10B). The results show that there are two ranges where the calibration curve produces good linearity. The first linear range with low concentration has a regression equation *y* = 2.25*x* + 5.78 with a correlation coefficient of 0.989, whereas the second linear range with high concentration has a regression equation *y* = 8.34*x* + 4.42 with a correlation coefficient of 0.991, where *y* represents the SPR angle and *x* represents the PAPPA2 concentration. Based on the calibration curve, this developed chip can used to quantitatively analyze the concentration of PAPP-A2 protein in spiked human plasma up to concentration of 0.01 pg/mL.

**Figure 11:**
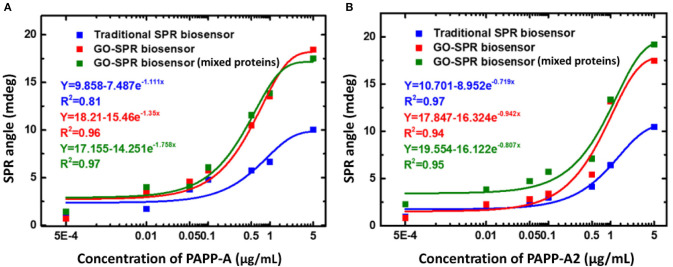
Surface plasmon resonance response curves and curve fitting equations of PAPP-A **(A)** and PAPP-A2 **(B)** measurement with the traditional SPR biosensor and GO-SPR biosensor. Figures **(A,B)** were reproduced from Fan et al. (2020) with permission from Dove Medical Press.

In 2020, Fan et al. (2020) detected PAPP-A and PAPP-A2 using GO-based SPR biosensor. To be able to detect PAPP-A and PAPP-A2, anti–PAPP-A and anti–PAPP-A2 were immobilized on the GO surface. Tests carried out in this experiment include the sensitivity and selectivity of biosensor in protein mixtures. Figure 11 shows the SPR response curves on traditional chip and GO-SPR chip for PAPP-A, PAPP-A2, and protein mixture sample (CK-19, HSA, hCG, CA 19-9, PAPP-A, PAPP-A2). Based on Figure 11A, when detecting PAPP-A, the SPR angle on the GO-SPR chip shows a better response than traditional chip. The same response also occurs when detecting PAPP-A2 (Figure 11B). This shows that the GO-SPR chip has better sensitivity than traditional chip. Furthermore, in the protein mixture sample, the SPR biosensor response to the detection of PAPP-A and PAPP-A2 showed a curve that was almost the same as the sample that was not mixed. Based on these data, it is proven that, in addition to sensitivity, biosensor selectivity also has good performance. The limit of detection in GO-based SPR biosensor in this experiment is 0.5 ng/mL.

The description above shows that, to be able to selectively detect a certain biomolecule, an appropriate bioreceptor is needed. Table 1 below shows a summary of research results on graphene-based SPR biosensors and the results obtained.

**Table 1:** Graphene-based SPR biosensor design, detection limits, and the resulting linear range.

**SPR system**	**Bioreceptor**	**Target**	**Limit of detection**	**Linear range**	**References**
Au/grapheme	α-thrombin aptamer	α-thrombin	0.05 nm	—	Wang et al., 2011
Au/graphene/gold nanostars	ssDNA	cssDNA	500 Am	—	Zagorodko et al., 2014
Au/grapheme	Cholera toxin antigen	Anticholera toxin	4 pg/mL	0.004–4 ng/mL	Singh et al., 2015
Au/grapheme	ssDNA	cssDNA	28 fM	—	Prabowo et al., 2016
Au/grapheme	Antifouling folic acid	Folic acid protein (FPA)	5 fM	5–500 fM	He et al., 2016
Au/GO-COOH	BSA	Anti-BSA	0.01 pg/mL	0.01–100 pg/mL	Chiu et al., 2017a
Au/GO	Peptide	hCG protein	0.065 nM	—	Chiu et al., 2017b
Au/SAM/GO/3ABA	Anti–gelactin-3	Gelactin-3	2 ng/mL	—	Primo et al., 2018
Au/GO-COOH	Lung cancer antibody	CK19 protein	0.001 pg/mL	0.001–100 pg/mL	Chiu et al., 2018
Au/GO-COOH	PAPP-A2	Anti–PAPP-A2	0.01 pg/mL	0.01–10.000 pg/mL	Chiu et al., 2019b
Au/GO	Anti–PAPP-A Anti–PAPP-A2	PAPP-A PAPP-A2	0.5 ng/mL 0.5 ng/mL	— —	Fan et al., 2020

Based on Table 1 above, to date, there are many graphene-based SPR chips that have been fabricated by researchers. In our laboratory, chips that have been successfully fabricated are graphene, GO, and GO-COOH–based SPR chips. On graphene-based SPR chip, graphene is usually immobilized on a metal surface using the chemical vapor deposition method and the method transferred by electrostatic adsorption. The advantages offered by this method include the ease of controlling the thickness of graphene. But the chip fabrication process is quite complex. In addition, the force produced between graphene and chip is van der Walls force or electrostatic force. This force is weak enough so that repeated detection becomes difficult to fulfill. The second chip is a GO-based chip. Graphene oxide can be immobilized on a metal surface by using a modified chemical covalent bond immobilization method. By using this method, the chip and GO bind with a very strong force, so it is not easy to fall off, and repeated detection is very possible. However, the thickness of GO grown by this method is very difficult to control. The last chip is GO-COOH–based chip. This chip can be fabricated using a modified chemical covalent bond immobilization method. The SPR chip fabrication process is moderate but produces SPR chip with very high biocompatibility. In addition, this method can make the wafer and GO-COOH a super strong binding force and not easy to fall off and can be used for repeated detection. But the thickness of the GO-COOH layer is not easily controlled. In other research groups, there are other types of SPR chips that have been successfully fabricated. Our analysis regarding the advantages and disadvantages of each chip is shown in Table 2.

**Table 2:** Graphene-based SPR chip, which has been successful in fabrication, advantages, and disadvantages.

**SPR system**	**Chip process**	**Repeatable detection**	**Thickness and precision of film making**	**References**
Au/grapheme	Complex	Difficult	Easy to control	Wang et al., 2011
Au/graphene/gold nanostars	Complex	Difficult	Easy to control	Zagorodko et al., 2014
Au/grapheme	Complex	Difficult	Easy to control	Singh et al., 2015
Au/grapheme	Complex	Difficult	Easy to control	Prabowo et al., 2016
Au/grapheme	Complex	Difficult	Easy to control	He et al., 2016
Au/GO-COOH	Moderate	Feasible	Difficult	Chiu et al., 2017a
Au/GO	Easy	Feasible	Difficult	Chiu et al., 2017b
Au/SAM/GO/3ABA	Easy	Feasible	Difficult	Primo et al., 2018
Au/GO-COOH	Moderate	Feasible	Difficult	Chiu et al., 2018
Au/GO-COOH	Moderate	Feasible	Difficult	Chiu et al., 2019b
Au/GO	Easy	Feasible	Difficult	Fan et al., 2020

## MoS_2_ Based SPR Biosensor

### MoS_2_ Properties and Their Potential in the SPR Biosensor

Two-dimensional MoS_2_ is an inorganic compound composed of molybdenum (Mo) and sulfur (S) (Das et al., 2015). This material is a semiconductor material with an ultrathin direct band gap and belongs to the transition metal dichalcogenide group. Molybdenum disulfide has characteristics similar to graphene; it is not affected by dilute acid or oxygen and is not reactive with other chemicals (Van Santen and Neurock, 2017). It also has the unique characteristics of electrical and photo-responsiveness of Shockley-type surface state properties. Therefore, MoS_2_ has been widely studied with regard to SP-enhanced photoluminescence, energy dispersion, integrated circuits, photosensitivity, and highly efficient emitter (Li and Zhu, 2015; Kalantar-Zadeh and Ou, 2016).

Lately, MoS_2_ has attracted the attention of researchers in the field of optical biosensors because of its high electron conductivity, tunable band gap, and high optical absorption efficiency. As a monolayer of MoS_2_ possesses a higher optical absorption efficiency (~5%) than that of graphene (2.3%) (Lopez-Sanchez et al., 2013), it can promote plasmon excitation through an efficient charge transfer between MoS2 and the thin metallic film (Kim et al., 2019). In addition, the large surface area and the presence of free sulfur atoms are typical features of MoS_2_, which make it a potential material for developing biosensing interfaces (Kaushik et al., 2019a). When MoS_2_ layers are deposited on metal thin films, the strong coupling can be induced at the metal/MoS_2_ interface because of the effective charge transfer and large electric field enhancement, which will result in increased SPR sensitivity (Hu et al., 2019). In addition, the MoS_2_ layer serves to inhibit the penetration of oxygen and water molecules to prevent oxidation of aluminum and silver metals (Xu et al., 2018; Kim et al., 2019).

### Effect of MoS_2_ on SPR Sensitivity

**Figure 12:**
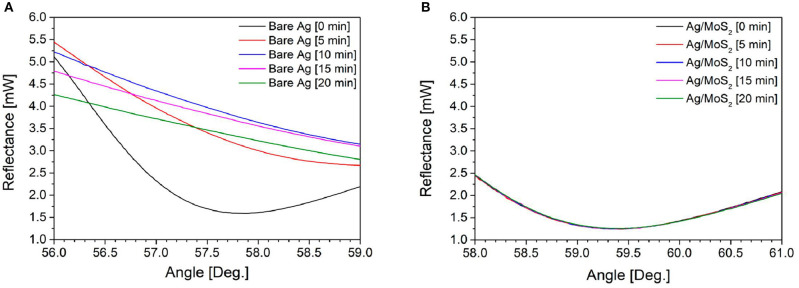
Surface plasmon resonance signals of **(A)** bare Ag and **(B)** Ag/MoS_2_ chip in water with laser irradiation. Figures **(A,B)** were reproduced from Kim et al. (2019) with permission from MDPI.

In 2019, Kim et al. (2019) used a thin silver layer to excite SP waves. They compared two different chips, namely, conventional chip (Bare Ag) and silver chip deposited with MoS_2_ (Ag/MoS_2_). Figure 12 shows the shape of the SPR signal measured every 5 min for 20 min on conventional chip and Ag/MoS_2_ chip. On conventional chip, the SPR signal shows a drastic change. This is presumably because the light that hits the prism triggers the oxidation of silver. Unlike the case with Ag/MoS_2_ chip, the SPR signal shows a consistent shape. This shows that the stability of the SPR chip on this chip is more stable than conventional chip. To determine the sensitivity produced, immunoglobulin G with a concentration of 600 nM is injected into each chip. Surface plasmon resonance angle shifts on conventional chip and Ag/MoS_2_ chip are 0.20 and 0.25°, respectively. Based on this study, it can be concluded that the presence of MoS_2_ monolayer has been shown to increase SPR stability and sensitivity up to 125%.

**Figure 13:**
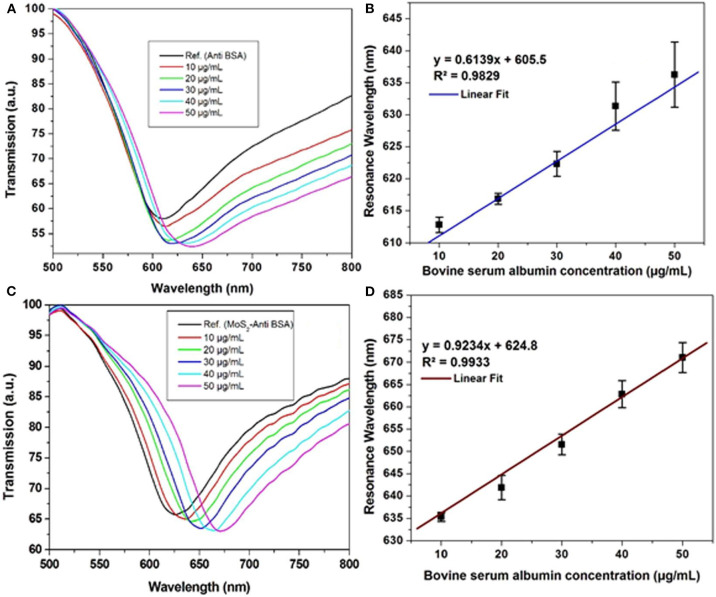
Surface plasmon resonance signal against BSA in phosphate-buffered saline (PBS) solution of the **(A)** optical fiber SPR biosensor without MoS_2_ and **(C)** developed SPR biosensor. Calibration curve of **(B)** Ab/gold/fiber; **(D)** Ab/MoS_2_/gold/fiber against varying concentration of BSA in PBS solution. Figures **(A–D)** were reproduced from Kaushik et al. (2019a) with permission from the Springer Nature.

In the same year, Kaushik et al. (2019a) investigated the effect of MoS_2_ on the gold surface using optical fiber-based SPR biosensor to study interactions between anti-BSA and BSA. Experiments were carried out by injecting BSA with different concentrations (10–50 μg/mL) on conventional chip and MoS_2_-based chip. The resulting SPR signal and calibration curve are shown in Figure 13. The slope on the MoS_2_-based SPR chip (0.9234) has a greater value than conventional chip (0.6139). In contrast, the limit of detection value on the MoS_2_-based SPR chip has a smaller value (0.29 μg/mL) when compared to conventional chips (0.45 μg/mL). These results confirm that the presence of MoS_2_ on the SPR chip is proven to increase sensor sensitivity (Kaushik et al., 2019a).

### Current Application of MoS_2_ Based SPR Biosensor

**Figure 14:**
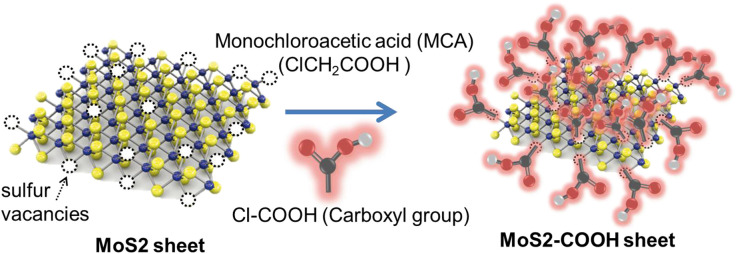
Synthesis of MoS_2_-COOH sheet with monocholoroacetic acid (MCA). Figure was reproduced from Chiu et al. (2018) with permission from Elsevier. Copyright 2018, Elsevier.

Development of MoS_2_ for SPR biosensors in a research group led by Professor Chiu started in 2018. Chiu and Lin (2018) developed MoS_2_ functionalized with the carboxyl group by utilizing the sulfur vacancy in MoS_2_. This vacancy will then be filled by Cl originating from Cl–COOH [–COOH modified with monochloroacetic acid (MCA)] to form a new sheet called MoS_2_-COOH sheet. Briefly, the experiments carried out are illustrated in Figure 14 below. The MoS_2_-COOH sheet is then applied to the SPR biosensor to detect protein antibodies. The results obtained indicate that the MoS_2_ functionalization with –COOH can strengthen the SPR biosensor response up to 3.1 times when compared to conventional SPR chip (Chiu and Lin, 2018).

**Figure 15:**
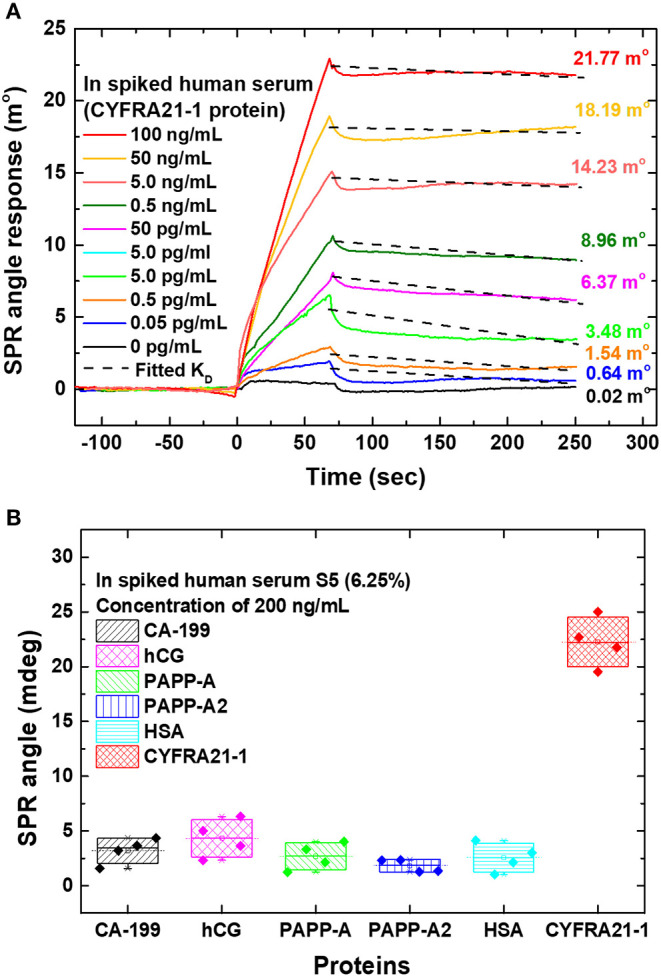
**(A)** The sensogram that shows the SPR response at different CYFRA21-1 protein concentrations. **(B)** Sensor selectivity for different types of proteins. Figures **(A,B)** were reproduced from (Chiu and Yang, 2020) with permission from Frontiers.

Chiu and Yang (2020) used a single-layer MoS_2_-COOH for signal amplification to detect lung cancer associated with the cytokeratin 19 fragment biomarker (CYFRA21-1) using an SPR-based biosensor. To detect CYFRA21-1, MoS_2_/COOH-based SPR chip was functionalized using lung cancer antibodies (anti-CYFRA21-1, TROMA-III). Figure 15 shows how biosensor performance and selectivity are produced. Based on Figure 15A, the greater the concentration of CYFRA21-1 (0 pg/mL−100 ng/mL), the greater the SPR angle shift. The largest SPR angle shift occurs with the highest concentration (100 ng/mL). Based on Figure 15B, biosensor selectivity also shows good results. The SPR angle shift on the CYFRA21-1 protein shows a much greater magnitude than other types of proteins (CA-199, hCG, PAPP-A, PAPP-A2, and HAS) (Chiu and Yang, 2020).

Research on the use of MoS_2_ in the SPR biosensor is still not widely done. At present, most studies carry out only computer simulations. Table 3 below shows a summary of research results on the MoS_2_-based SPR biosensors in our research group and other groups conducting the same experimental study.

**Table 3:** Molybdenum disulfide-based SPR biosensor design, detection limits, and the resulting linear range.

**SPR System**	**Bioreceptor**	**Target**	**Limit of Detection**	**Linear range**	**Ref**.
Au/MoS_2_/AuNPs	Micro RNA-141	Micro RNA-141	0.5 fM	-	Nie et al., 2017
Au/MoS_2_-COOH	Anti-BSA	BSA	1.45 pM	14.5 – 725 nM	Chiu and Lin, 2018
Ag/MoS_2_	-	immunoglobulin G	-	-	Kim et al., 2019
Au/MoS_2_	Anti-BSA	BSA	0.29 μg/mL	-	Kaushik et al., 2019a
Au/MoS_2_	*E. coli* monoclonal antibodies	*E. coli*	94 CFU/mL	-	Kaushik et al., 2019b
Au/MoS_2_/AuNPs	Goat-anti-mouse IgG	Mouse IgG	0.06 μg/mL		Zhao et al., 2020
Au/MoS_2_-COOH	Anti-CYFRA 21-1	CYFRA 21-1	0.05 pg/mL	0.05 pg/mL – 100 ng/mL	Chiu and Yang, 2020

*CFU: Colony forming units*.

To date, MoS_2_-based chips fabricated by our research group have been successfully applied to detect protein antibody and CYFRA 21-1. The two chips that are fabricated are MoS_2_ chip that are functionalized with COOH. COOH can bind strongly to MoS_2_ due to strong bonds due to the presence of sulfur vacancies in MoS_2_. Molybdenum disulfide–COOH cannot be immobilized directly on a metal surface. Therefore, thiol- and amine-group self-assembled monolayers of cystamine (Cys) function as a bridge for immobilization of MoS_2_-COOH sheets on metal surfaces. A complete analysis related to the SPR chip fabrication process, the advantages, and disadvantages are shown in Table 4.

**Table 4:** Molybdenum disulfide–based SPR chip, which has been successful in fabrication, advantages, and disadvantages.

**SPR system**	**Chip process**	**Repeatable detection**	**Thickness and precision of film making**	**References**
Au/MoS_2_/AuNPs	Complex	Feasible	Difficult	Nie et al., 2017
Ag/MoS_2_	Easy	Difficult	Difficult	Kim et al., 2019
Au/MoS_2_	Easy	Difficult	Difficult	Kaushik et al., 2019a
Au/MoS_2_	Easy	Difficult	Difficult	Kaushik et al., 2019b
Au/MoS_2_/AuNPs	Complex	Difficult	Difficult	Zhao et al., 2020
Au/MoS_2_-COOH	Complex	Feasible	Difficult	Chiu and Lin, 2018
Au/MoS_2_-COOH	Complex	Feasible	Difficult	Chiu and Yang, 2020

## Conclusion and Future Research

Based on the above review, it can be concluded that 2D nanomaterial (graphene and MoS_2_) has been proven experimentally to increase the sensitivity of the SPR biosensor. Several experiments conducted in our laboratory have shown that GO or MoS_2_-based SPR biosensors that are functional with the carboxyl group have been shown to increase sensor sensitivity. In several different detection applications (anti-BSA, anti-PAPPA2, CYFRA 21-1, and CK19), the limit of detection shows the same level that is at the pg/mL level. This result can be used as a foundation for a wider diagnostic application. Although, experimentally, graphene-based SPR biosensors have been widely used for a variety of applications, the mass production of SPR chips still needs further research, especially those related to efficiency in the fabrication process of SPR chips. The shape, size, number of layers, electronic band gap structure, purity, and graphene defects that grew in the experiment are all uncertain. These properties can influence the conductivity of the SPR chip, interaction with biomolecules, and fluorescence quenching causing the performance of graphene-based SPR biosensors to be different for each fabrication. For MoS_2_-based SPR biosensor, so far it has not been possible to place monolayer of MoS_2_ uniformly on a larger surface area.

A high sensitivity detection device is needed as a pre-cautionary measure before the spread of disease in the human body. For this purpose, the combination of some 2D material can be one of the topics that is widely studied in the next few years. For example, the combination of MoS_2_ and graphene has been shown to increase SPR sensitivity through a simulation approach. But so far, there have been no publications containing experiments on this structure. Not only in 2D graphene and MoS_2_ material, but also other types of 2D material such as WSe_2_, MoSe_2_, WS_2_, and black phosphorous can be alternatives.

## Author Contributions

DN wrote the manuscript and performed the overall editing of the manuscript. N-FC provided guidance for the manuscript setups and manuscript structure. N-FC and Y-HW oversaw the project and performed the overall editing of the manuscript. The manuscript was written through contributions of all authors. All authors have given approval to the final version of the manuscript.

## Conflict of Interest

The authors declare that the research was conducted in the absence of any commercial or financial relationships that could be construed as a potential conflict of interest.
